# The Economic Impact of the SARS Epidemic with Related Interventions in China

**DOI:** 10.3390/ijerph192013263

**Published:** 2022-10-14

**Authors:** Haoyu Wang, Yishan Zhang, Yingying Qin, Chao Chen, Beason Richard

**Affiliations:** 1 Center for Quantitative Economics, Jilin University, Changchun 130012, China; 2 School of Economics and Management, Anhui Normal University, Wuhu 241000, China; 3 Alberta School of Business, University of Alberta, Edmonton, AB T6G 2R3, Canada

**Keywords:** macro-economics, severe acute respiratory syndrome, GDD model

## Abstract

Epidemics represent a threat to human life and economy. Meanwhile, medical and non-medical approaches to fight against them may result in additional economic shocks. In this paper, we examine the economic impact of the 2003 SARS outbreak in China and associated government policies. Although the epidemic caused a substantial economic loss in the short term, the interventions for medical purposes positively impacted the economy of the severely affected regions through the increase in investments such as other fiscal stimuli. There is strong and robust evidence suggesting that the SARS epidemic and its associated countermeasure policies boosted local output by around 4% and industrial production by around 5%. The positive growth was mainly derived from the increase in investment and government activity, especially government expenditure. Besides that, lagged impacts were particularly pronounced to the economic system and lasted for longer even than the epidemic period in a biological sense. We attribute this to the relatively aggressive stance of policymakers in the face of the epidemic situation.

## 1. Introduction

Infectious disease outbreaks, such as Black Death, 1918 flu, and more recently, bird flu (H5N1) and COVID-19, are serious threats to human life. At the same time, it is problematic for the economy and society, imposing a heavy public health burden. Thankfully, increasing approaches have been developed by modern medicine and epidemiology to control or eradicate diseases, but limited attention has been put on the economic impact of these policies designed for medical purposes. To reach that goal, we used the Severe Acute Respiratory Syndrome (SARS) epidemic in 2003 in China for our study. The reason being that at the time it was an unknown deadly infectious disease which was effectively controlled by the government. With this background our paper seeks to assess the economic impact of the SARS epidemic under relevant interventions made by China’s government during the 2003 outbreak.

Several approaches to assess the economic impact of epidemics have been developed in the past decades. The orthodox approach has employed several cost-of-illness measures or economic burden estimations, including medical costs, non-medical costs and productivity losses and other costs caused by the relevant disease [1]. Another example is de Francisco, Shapovalova et al. [2], which reviews 62 studies that assess the economic burden or cost of influenza. Besides, some works used tendency analysis to estimate the economic indicator’s response to the epidemic [3,4].

Some studies focus on the question of how epidemics affect the economy. An obvious mechanism is that the epidemic would permanently affect labor supply [5], which leads to a relative scarcity of capital and increases the natural rate for more than 40 years [6]. There are several indirect mechanisms that have been examined as well [1]. The public’s perception of the risk of becoming infected or fear of infection is likely to reduce unnecessary contact between people [7]. Consumption would be distorted when exposed to the disease, like a “random tax” on the public’s welfare [1]. Some works, especially survey analysis, found that consumption will shrink during epidemics [8], or the epidemic alters consumption patterns [4]. Recent works hold the point that the epidemic shock would increase uncertainty in the financial market and economy, leading to a series of economic consequences [9,10,11].

Due to differences in the diseases, and under different medical conditions and public health policies, the economic impact of epidemics changes from period to period, city to city and country to country. The SARS epidemic whose characteristics are similar to COVID-19, such as the form of spread, mortality and so on, drives public health policymakers to impose policies on controlling the epidemic, which may provide some empirical evidence to help design recovery plans to deal with the economic impact of the COVID-19 epidemic. Some studies focus on the economic impact of the SARS epidemic. Several industries, such as tourism, leisure activities and transportation, have been highlighted as the industries with the worst losses [4,12]. Other researchers modelled the SARS epidemic shock on the economy [13]. Recent research compares the impact of the SARS epidemic and the COVID-19 epidemic in China in a Computable General Equilibrium model informed by an epidemiological model [14]. SIR models that could simulate the diffusion of the virus were introduced into model to estimate the socio-economic impact [15] or simulate public health policies [16].

In summary, there is a large body of literature documenting the instantaneous loss or economic loss in some specific industries which are supposed to be the worst hit during the epidemic period of SARS, but there is little research using long-term macro empirical evidence. Besides, compared to other epidemics, the SARS epidemic was caused by an acute and fatal disease that was brought quickly under control by public health policies in China. The lack of long-term analysis led to a lack of understanding of the economic impact of the policies.

The rest of the paper is arranged into several sections: the rest of this section will review what happened regarding the SARS epidemic in China and what China’s government did during the SARS epidemic. Section 2 constructs an epidemic shock model from the producers’ perspective, considering the epidemic and relevant policies as a whole, and designs a corresponding empirical strategy that uses the General Difference-in-Difference (GDD) approach to estimate the economic impact. Section 3 provides the main results of our work with related robustness checks. The shock mechanism is also identified statically and dynamically in Section 3. At the end of the section, it is extended to the long term and the epidemic reaction mechanism’s economic impact is tested. Section 4 discussed and Section 5 concludes.

### Background: SARS in 2003 in China

At the end of 2002, the first case of severe acute respiratory syndrome (SARS) virus was identified in Shunde City of Guangzhou province in China. The virus subsequently spread to the whole country and then spread to Asia as well as western countries. Starting from 10th February, the Chinese government reported infected cases and the virus to World Health Organization (WHO). Thousands of people were infected in China by the epidemic and hundreds of them died from infection.

Since the beginning of April 2003, the Chinese government activated epidemic control mechanisms, including travel restrictions, fever testing, the establishment of isolation hospitals and patient isolation. Meanwhile, the government encouraged citizens to use face-masks and other disinfection measures to contain the virus’s spread, which led to shortages of masks and disinfectants at that time. Primary schools and secondary schools across the country were suspended, while some universities in Beijing closed their campus. In some hard-hit areas, some factories were closed. It was not over until 22nd May when Beijing reopened its schools.

In China, 24 out of 31 provinces of the mainland were infected. Macao, Hong Kong and Taiwan also had found cases of infection. Between each area across the country, travel restrictions were imposed to the high-risk areas. Simultaneously, the government tried initiatives to reduce public transportation usage through media and set up temperature detectors at airports and railway stations.

Many studies have proved that the SARS epidemic causes a sharply negative economic shock instantaneously. Beutels et al. [4] show that leisure activities, local and international transport and tourism were hit by SARS in May 2003, and led to an irrecoverable loss at about $1.4 billion. Keogh-Brown & Smith [17] assess the macro-economic loss caused by the SARS epidemic suggesting the epidemic had a catastrophic effect on the global economy. However, limited attention is put to the recovery after the outbreak.

## 2. Materials and Methods

### 2.1. Theoretical Perspectives

From a purely economic point of view, Karlsson et al. [18] developed a model based on the Lucas model to hypothesize that the 1918 flu pandemic was a labor supply shock to the economy while it left physical capital intact. Karlsson’s model assumed the contagion would cause a severe loss on labor supply, which is the primary source of the epidemic’s economic impact. However, thanks to the development of medical technology and epidemiology, human beings have more approaches and interventions to contain contagions since the 21st century. The procedures and policies, including isolation and trace surveys, are widely applied in the prevention of severe infectious diseases, reducing infection rates during the pandemic, such as during H1N1, COVID-19 and SARS.

Comparing the 1918 flu pandemic to SARS, the 1918 flu pandemic caused an estimated 500 million cases infected worldwide, about 10 million of whom died [19], which affected almost half the world’s population at that time. Compared to that, in 2003, only a low proportion of labor supply, less than 0.02% of the Beijing City population where the virus infected most, were infected or killed by SARS in China, while it caused severe short-term economic consequences.

We will briefly describe a simple supply side model to explain the economic consequence of these policies. Let us start our model from the Cobb–Douglas function. In the labor market, the isolation policy and travel restrictions will create a short-term labor supply shock, while the epidemic may slightly hit the labor population permanently under the effective regulation. There are two reasons that we assume the labor shock will lead to a temporary proportionate loss of labor. First, the scale of infection is related to population density. The higher the population density is, the higher risk of infection is. Infectious diseases are more likely to spread in densely populated areas, both in terms of speed and scale, leading to a more significant loss of labor. Second, the regional government in the densely populated locations is more sensitive to the number of people infected. Considering the socioeconomic impact of the epidemic, an effective government would put in some measures to control the epidemic in time. Because it is more challenging to control disease in a region with a large population, the government tends to put in more strict regulations in such areas, including spending more on epidemic prevention, employing more workers to survey and purchase more medical resources. Following this assumption, the coefficient of labor loss in the epidemic could be introduced to the production function in the following form:

(1) $$Y_{i,t}=A_{i,t}K_{i,t}^{\alpha }{\left[L_{i,t}\cdot \left(1-D_{L}\right)\right]}^{\beta }$$

where $Y_{i,t}$ donates the output in region *i* at time *t* and $K_{i,t}$, $L_{i,t}$ represent the capital and labor factors put into production. We do not assume the specific form of factor shares α and β, but assume they are not specific by region or time. Let $\left(1-D_{L}\right)$ as labor loss coefficient catch the short-term impact of the epidemic on the labor market, if and only if at time T during the epidemic outbreak, $D_{L}$ is not 0. The logarithmic difference equation can separate the economic effect on labor supply of the epidemic:

(2) $$y_{i,t}=a_{i,t}+\alpha k_{i,t}+\beta l_{i,t}+\beta ln\left(1-D_{L}\right)$$

where the lowercase is representing the logarithmic form factors and output. In this model, $a_{i,t}$ is the residual of the growth accounting or Solow residual, while some paper uses the indicator to represent the total factor productivity (TFP).

Concerning the capital factor, some of the government’s behavior may affect the capital input during the epidemic. On the one hand, government purchases drive government capital into the production process in the short term. Due to the epidemic, medicines, medical instruments and other facilities to prevent infectious diseases cannot meet society’s demand, the government, a massive buyer for these products, orders the products and hands them out to citizens while these are produced. Facing the shock, the unprepared governments sometimes issue special bonds to raise money for the facilities and cut spending on some industries or benefits for residents, which is equivalent to driving capital transfer cross-industry and cross-period to the relevant supply chain. Because this kind of purchase is direct to the enterprise, it does not directly affect the capital market’s interest rate. Another similar government behavior is that the recovery plan is also aimed at inducing investment. However, additional investment for the above purpose would be controlled by the capital indicator in our model.

On the other hand, lockout policies on factories or exchanges would be a retardant to capital. Suppose the epidemic is out of control in a region. In that case, the central government may lock down the city to prevent infection in other areas, such as Wuhan in 2020. Whatever the lockout policies or the stricter lockdown policies, the result is a decrease in the capital put into the supply side, and then, reduces total output.

The two mechanisms are also related to capital scale: on the aspect of purchases, under the uncertainty of the epidemic, a rational government attempting to minimize civilian deaths will maximize its purchases, which is constrained by the size of its finances relative to regional capital scale. On the other side, it is related to the production scale and capital market size and the capital loss caused by lockdown or lockout policies in the production process. Therefore, we define a capital change coefficient as having a similar form to the labor loss coefficient. We can describe the impact of the epidemic on the economy, ρ, by:

(3) $$y_{i,t}=a_{i,t}+\alpha k_{i,t}+\beta l_{i,t}+\rho \;\;\rho =\alpha ln\left(1-D_{K}\right)+\beta ln\left(1-D_{L}\right)$$

In addition to the direct impact of the epidemic, public health intervention during the epidemic period and the recovery plan in the post-epidemic period also had a profound impact on the economy. During that period, the government transferred capital to the market in various ways: such as the expansion of government purchases for controlling the epidemic, including purchasing medical resources and employing medical workers and social workers, would expand demand, and thereby stimulating investment and employment through the increase in the rate of interest and wages, which may mitigate the negative shock of the epidemic. Alternatively, policies might result in a reduction of the cost of capital and human resources via tax reductions. Obviously, the scale of impact depends on the scale of the epidemic in the policymakers’ area of administration and is limited by resource constraints.

In the Chinese case during 2003 the central government allocated RMB 2 billion (regional governments provided an additional RMB 3 billion) to set up a SARS epidemic prevention and control fund. The National Development and Reform Commission of China allocated treasury bonds to the construction of epidemic prevention facilities on a scale of about RMB 12.6 billion.

Additionally, China’s State Council, Ministry of Finance and local governments have introduced various tax and fee reduction policies, including income tax reductions, city preservation and development tax, general government funds and other measures. These demand management policies and the tax and fee reduction policies expanded the production process and output through the expansion of investment and employment mitigating the shock of the epidemic.

Based on that, the following equation is used to capture the economic impact of government activities,

(4) $$y_{i,t}=a_{i,t}+\alpha k_{i,t}+\beta l_{i,t}+\rho \;\rho =\alpha ln\left(1-D_{K}\right)+\beta ln\left(1-D_{L}\right)+\left[\alpha ln\left(1+D_{G}^{K}\right)+\beta ln\left(1+D_{G}^{L}\right)\right]$$

However, the *D* coefficients defined above cannot be observed directly. It is hard to estimate how many potential migrants were restricted from work or forced to cancel their travel plans for other relevant reasons. Similarly, there are no suitable indicators to measure how much capital would be utilized in production if SARS had not occurred or how much cash flow was blocked owing to the health emergency.

To get at the effects of the epidemic and relevant policies, we introduce a natural experiment method to estimate it. We regard the outbreak as a treatment to regions and use some comparable samples to the affected regions. The following equation compares the output between the treatment group (*i*) and control group (*j*),

(5) $$y_{i,t}-y_{j,t}=\left(a_{i,t}-a_{j,t}\right)+\alpha_{i}\cdot k_{i,t}-\alpha_{j}\cdot k_{j,t}+\beta_{i}\cdot l_{i,t}-\beta_{j}\cdot l_{j,t}-\rho \;\rho =\alpha ln\left(1-D_{K}\right)+\beta ln\left(1-D_{L}\right)+\left[\alpha ln\left(1+D_{G}^{K}\right)+\beta ln\left(1+D_{G}^{L}\right)\right]$$

The treatment effect, ρ, (in this case means the impact on output) could be estimated under the assumption that the Solow residual is equal between the two groups, which is,

$$H_{0}:a_{i}-a_{j}=0$$

However, since TFP and shocks are still unobservable (though several methods exist to estimate TFP, which are positively related to inputs), it is impossible to test the above hypothesis. Therefore, we incorporate the time dimension to estimate and test the theory, although it may lead us to overestimate the time impact. Therefore, we use the Difference-in-Difference (DD) model to catch the effect among the time series between the two groups. Because the treatment effect, ρ, is inseparable from the Solow residual, it is a good idea to use the DD model to compare the residual between the outbreak period of SARS in China and the non-outbreak period so that we can extend the hypothesis to the following form,

$$H_{0}^{1}:a_{i,t}-a_{j,t}=0,\;t\notin t_{outbreak}\;H_{1}^{1}:a_{i,t}-a_{j,t}=\rho ,\;t\in t_{outbreak}$$

If the samples between the treatment group and control group are comparable under the non-outbreak period, the residuals do not differ, while the division between the two groups is equal to the treatment effect ρ. The regions between groups may not be comparable perfectly, but if the difference between the regions is constant, we could estimate the treatment effects by the form,

$$H_{0}^{2}:a_{i,t}-a_{j,t}=c,\;t\notin t_{outbreak}\;H_{1}^{2}:a_{i,t}-a_{j,t}=c+\rho ,\;t\in t_{outbreak}$$

where *c* is the constant difference between the two groups unrelated to the epidemic.

### 2.2. Empirical Strategies

To get the impact of the SARS epidemic and related interventions, the impact can be estimated by the standard DD model as follows,

(6) $$y_{i,t}=\rho \cdot tre\cdot post+\gamma_{1}tre+\gamma_{2}post+Xb+\varepsilon_{i,t}$$

where, $y_{i,t}$ designates the outcome, tre is a dummy variable that distinguishes whether SARS hit the region, and post is another indicator that takes on the value one in the post-epidemic period. Control variables X control for the input variables, in this case, we control capital and labor factors. The random disturbances, $\varepsilon_{i,t}$, are time-varying and region-varying. However, under the standard DD model, the post term takes one for all the period after the outbreak or other disasters happen. The *post* term is able to capture structural changes, (such as the economic effect of minimum wages) but would confound the shocks with the new steady state, such as epidemics or other disasters. Therefore, following Belasen & Polachek’s [20] specification of research on hurricane impact, we differentiate the equation above among the time series to focus on the epidemic’s economic effects. Moreover, the tre term and post term give too strong but unnecessary constraints to the regression and neglect the control of regional heterogeneity and macroeconomic events. By loosening these constraints, we use regional fixed effects, $\eta_{i}$, and time fixed effects, $\eta_{t}$, to replace the original two to make the estimation more accurate, while region fixed effects control for the permanent heterogeneity across regions and time fixed effects control for year-specific shocks to all the regions in our samples. With these adjustments, the model takes the following form and can be estimated for the impact of the SARS epidemic,

(7) $$\Delta y_{i,t}=\rho \cdot tre\cdot shock+\Delta Xb+\eta_{i}+\eta_{t}+\varepsilon_{i,t}$$

where the term shock is the difference of the term post which is a dummy variable that takes one during the epidemic outbreak. Meanwhile, under this specification, the unit root, if there were one, would be mitigated.

### 2.3. Data Sources

#### 2.3.1. Economic Indicators

For consistency of theory and estimation, we choose output, population and capital stock of each region of China as the economic indicators for analysis. The spatial dimension of the primary estimation in this paper is based on China’s provincial administrative division because the intervention decisions were designed by the provincial and central government, but we also report some vital estimates from city-level data to check its robustness. On the time dimension, this paper uses annual data for estimations from 2000 to 2016 (for province-level data) and from 2000 to 2006 (for city-level data), because China’s National Bureau of Statistics adjusted its statistics plan at the city level after 2006.

Gross regional product as well as industrial output were chosen as proxy variables for production in the model. The labor factor selects the number of permanent residents in the region as the proxy variable. There are many discussions on the estimation of capital stock, especially on the selection of initial value and depreciation rate. This paper chooses an estimation of capital stock widely recognized by Chinese researchers, developed by Shan [21], and extends the data to 2016 with the sequence of total investment in fixed assets as an investment. We use investment data to indicate the difference in terms of capital, due to measurement errors from the initial value at the city level. To avoid the dispute on the estimation method of capital stock, the results estimated with the investment indicator are not reported but are similar to the corresponding results estimated by capital stock. All the data for these indicators and their estimation process are from the China Statistical Yearbook published by the National Bureau of Statistics of China.

#### 2.3.2. Epidemic Indicator

As we mentioned above, the SARS epidemic outbreak began in 2003 in China, so let shock indicator, shock, take the value one only when t=2003. When it comes to the description of the SARS epidemic, we use the cumulative clinical diagnosis sequence of SARS published by the Ministry of Health of the PRC (now the National Health and Family Planning Commission since 2013) on 31st May 2003. We use a cutoff point at 100 cases dividing the samples into two groups (where the cutoff point is set between Jilin province: 35 cases and Tianjin City: 175 cases). Therefore, this paper uses the standard that “whether the number of infected people in the region exceeds 100 cases” as the treatment standard to divide the samples into two groups: treatment group and control group, or in other words, severe epidemic group and slight epidemic group. Under this standard, six provincial administration regions are classified into the treated group, including Beijing city (2521 cases), Guangdong province (1511 cases), Shanxi province (450 cases), Inner Mongolia Autonomous Region (284), Hebei province (215 cases) and Tianjin city (175 cases).

## 3. Results

### 3.1. Empirical Analyses

Table 1 shows the estimation results of Equation (7) where panel A presents the treatment effect on all the sectors while panel B focuses on industrial sectors. Column (1) provides the result without any controls except province fixed effect and year fixed effect, indicating that the regions with severe epidemic had achieved about 4% higher economic growth than regions with slight shock. Column (2) reports the estimation of treatment effect with control variables, suggesting a significant positive treatment effect at a 1% significance level. In comparison, panel B reports a higher positive treatment effect on industry at a 10% significance level. In order to avoid statistical inference bias caused by heteroscedasticity or residuals following non-normal distribution, Column (3) reports the estimation results with standard bootstrap errors. The results are robust with controlled by investment.

Further, Columns (4)–(7) report the analysis results using city-level data. We use the same standard to define the term tre by “whether the city belonged to a province where the SARS epidemic infected more than 100 cases in 2003.” We choose the standard for two reasons: On the one hand, the Ministry of Health did not publish data on SARS infection at the city level at that time. On the other hand, the interventions were designed by the health department of each provincial government or the health bureaus of the province-level municipalities. Thus, cities in the same province were affected similarly by the policies.

Considering the more serious heteroscedasticity problem in city-level data, columns (4)–(6) use robust standard errors for statistical inference. Based on the estimation of Equation (7) in Column (4), Column (5) replaces the province fixed effects with a city fixed effect. Column (6) further controls for capital and labor inputs. Since there is no reliable estimate of the initial value and depreciation rate of the capital stock at the city level in China, we use the investment as a proxy for the difference of capital stock, ΔK. Concerning heterogeneity, even though we control observable input variables in regression, we address this issue by focusing on the observations on common support of the propensity score in Column (7), which linked the sample to cities between groups that have similar observed input or size. The parameter estimated by PSM-DID is similar to estimation by robust OLS, whether on total output or industrial output and remains significant.

Compared to the whole economy and non-outbreak periods, industry had achieved better growth during the epidemic, judging from the two pieces of evidence in Table 1. On the one hand, the estimated effect of the epidemic and its related policies on industrial production are about 1% higher than the corresponding results on total output, whether at the province level or city level. Another result is that the treatment effects among cities are slightly lower than it in provincial analysis. City-level data are weighted equally to the metros with industrial agglomeration and the small cities, while province-level data emphasis the city with larger output within the province. Therefore, provincial data show more statistical characteristics of large cities, especially on industrial production. Another concern is whether the direct effects of the outbreak or the economic impacts of its related policies were slighter in the small cities or sparsely populated countryside.

### 3.2. Robustness Checks

In the last section, we provided some evidence on the SARS epidemic’s impact and relevant policies. It may be beyond common sense or economic intuition that the policies or the disease itself had significantly accelerated economic growth at about 4% or industrial production growth at approximately 5%. We provide some further robustness checks to support the analysis above in this subsection for three aspects: sample length, de-inflation and variable selection. The results of robustness checks are reported in Table 2.

Firstly, we confirm that the treatment effect is not sensitive to the length selection of the dataset. A too-long sample length may cause some unobservable trend-related confounding factors to be introduced to the analysis, while a too short sample length would result in an insufficient description of common trends. Columns (8)–(10) in Table 2 present the longer and shorter sample length selection than it in Table 1, controlling for time and province fixed effects. The first four columns select the data length for 20 years from 1996 to 2016, while the last four estimations have covered the period between two global economic crises from 1999 to 2007 to avoid the different responses of the shocks between the groups if it did. The estimated coefficients stay significant and positive. Meanwhile, we find some changes between different datasets both on estimated coefficients and significance levels, suggesting that the effects may be time-varying or have a dynamic response mechanism that we will examine In the next section.

Secondly, price changes were not the real source of the treatment effects described in this paper. Some papers suggested that the price level or inflation will change significantly during the epidemic period [22,23,24], which may hide the treatment effects on the real economy. We address this issue by using the logarithmic gross regional product index at constant price published by the national bureau of statistics of China as the proxy variable for output, reporting the results in Column (9) and (13) with different sample length. The results stay significant and positive but decrease slightly. It suggests that price had been affected by epidemic shock but was not the only source of the treatment effect.

The last concern is that the use of control variables still cannot control the bias caused by the scale of regions. Besides, the collinearity between the treatment effect and size might lead to bias. Therefore, we take an alternative indicator, output per capita, to be used in the estimation. The results in Column (10) and (14) present similar results to Column (8) and (12). We also provide the result on the GRP index at a constant per capita at a constant price in Column (11) and (15). By stripping out the currency effect, the treatment effect declines but remains significant. Meanwhile, it is robust at the per capita level.

### 3.3. Mechanism Identification

Contrary to economic intuition, the previous analysis has concluded that the epidemic promoted economic growth from the supply side. However, it is difficult to decompose the Solow residual to find out which part of the economic system positively responded to epidemic shocks, which led us to identify the specific mechanism of the epidemic’s impact on the economy from a demand-side view. Therefore, this section will start from the macroeconomic identity to decompose economic growth to explore the specific mechanisms of how it leads to economic acceleration.

As mentioned above, observing changes in the estimated coefficients over different periods, we noted that the epidemic shock might have not only a short-term effect but also duration. The second subsection will discuss a dynamic epidemic mechanism on the economy to explore the persistence and time-varying treatment effect. In addition, we will examine the common trend hypothesis and comparable hypothesis in a dynamic model.

#### 3.3.1. Growth Decomposition

According to the macroeconomic identity, output consists of consumption, investment, government purchase and net exports. According to that, we use similar empirical strategies to test the responses in various parts of output to the epidemic shocks. However, due to different statistical definitions, the National Bureau of Statistics of China does not provide data according to these national account components. This paper uses some proxy indicators to describe various parts of the economy.

We use the total retail sales of consumer goods to describe changes in consumption; we use total investment in fixed assets in the whole region to represent investments; use the general budget expenditure and income of the local government and its difference, the fiscal deficit, to manifest the local government activities and relevant policies used in the period of the epidemic; use freight traffic and passenger traffic to measure the trade and other inter-region relationships. All these data are from the National Bureau of Statistics of China. It is worth mentioning that the investment data calculated by China Statistics Bureau includes not only private sector investment but also public investment. The data began from 1996 when Chongqing was promoted to a provincial administrative region to 2008 when the financial crisis broke out. The decomposition results are reported in Table 3 without any other control except for the provincial fixed effect and year-specific fixed effect. The following conclusions can be drawn from the table.

First of all, consumption and trade were not affected significantly by the epidemic or regulation in 2003. The results in Column (16), (21) and (22) show that consumption and inter-regional economic activities were slightly reduced but not significant. Although some previous works found that the epidemic severely hit trade and tourism, it recovered soon and rapidly covered almost all the negative shock during the year. It seems that consumers impacted by the disease did not cancel their consumption plan but postponed it. The conclusion supports the point held by Keogh-Brown & Smith [17] that there were indeed some large short-term losses in China, but its rapid return to normality after which consumer confidence returned, stock replenished and purchases which were delayed by the disease rebounded. With the aspect of passenger traffic, some previous works examined international travel to China hit by the SARS epidemic, but in terms of domestic traffic in this paper, the negative effect caused by the epidemic was not significant. A possible reason is that the development of tourism in China was still in its infancy in 2003 when tourism’s total revenue only accounted for about 3% of GDP. Passengers for tourism purposes account for only a small proportion of all traffic. Therefore, although tourism was hit severely, it still led to a small negative impact on traffic or the economy.

Secondly, investment was the main source of the positive treatment effect on the economy during the SARS epidemic. Column (17) in Table 3 reports that investment growth in severely affected areas was significantly higher than it in other areas. The policies, whether for controlling the virus or aiding the economy, led to larger investments. As for Chinese data for investment, this series consists of not only the private sector but also the government, which may make it difficult for us to clearly distinguish the purpose of the policies for the primary source of positive treatment effect. Government spending on medical supplies would enhance investment, while policies aimed at saving the economy would also stimulate investment recovery. However, we may find additional evidence in government activities to distinguish them.

Thirdly, government activities were another source of the epidemic’s economic impact. The treatment effect test on financial expenditures and deficit is significantly positive, while financial revenue decreased slightly but not significantly as reported in columns (18)–(20). As mentioned above, the non-medical public health interventions, including isolation, distribution of medical supplies, the establishment of infectious disease hospitals and so on, were mainly in the form of government purchases, in other words, government spending. Other policies aimed at restoring the economy related were implemented in the form of tax and fee discounts, which related to financial income. According to the results in Table 3, it is obvious that the former was the more likely cause of the positive impact of the SARS epidemic. It was epidemic control and prevention policies rather than recovery plans pushing accelerated economic growth.

#### 3.3.2. Dynamic Mechanism Identification

Jordan, Singh and Taylor [6] reviewed 19 of the great historical pandemics since the 14th century and found that the pandemics had effects for decades on the returns to assets and the real natural rate of interest. It is still doubtful whether the SARS epidemic and its relevant policies would have a long-run economic impact by finding their treatment effect changed slightly under different sample lengths.

It is also necessary to test the hypothesis we defined above to confirm that the two groups were comparable on Solow’s residual without the epidemic’s shock. We have tested the hypothesis and judged whether there are any significant differences between the two groups before the epidemic. The test also can be used as proof of common trends.

For these purposes, we introduce a set of counterfactual terms to Equation (7) before and after the time of the SARS epidemic outbreak, that is, we set the *did* estimator in all the years in the sample as the following form,

(8) $$y_{i,t}=\sum_{year=0}^{T}\rho_{t}\cdot shock_{t}\cdot tre+X_{i,t}\theta +\eta_{i}+\eta_{t}+\varepsilon_{i,t}$$

where the terms $shock_{t}$ are a series indicator taking the value one if and only if the indicator t=year, and the term $\rho_{t}$ still represents the treatment effects but has some different meaning: when the time indicator t<2003, these terms test the expected treatment effect which was supposed to be 0, in other words, these test the comparable and common trend hypothesis; while for the time indicator t=2003, the terms test the treatment effect; while the time indicator t>2003, the terms test the post-epidemic treatment effect and how long it lasts.

**Figure 1:**
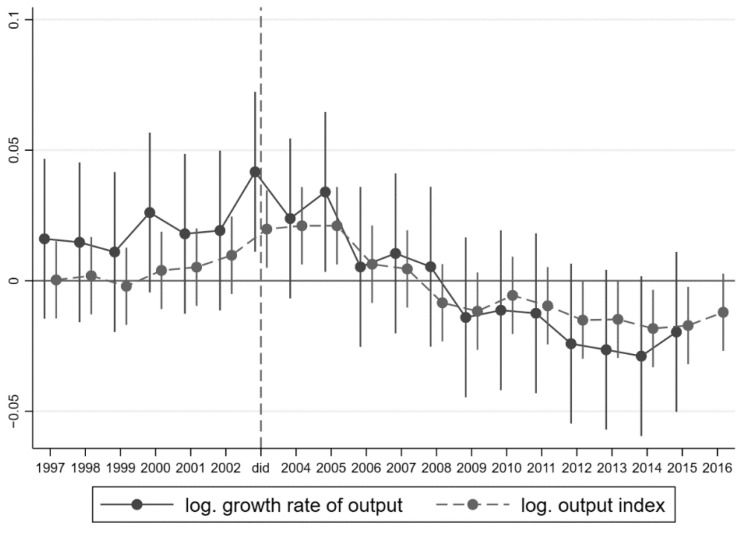
Dynamic Mechanism Identification.

Table 4 shows the results of using Equation (8) for testing. The results report that all the variables were compared with the common trend before the epidemic at the significance level of 99%. Specifically, Table 4 reports the test results of comparable hypothesis in Column (23)–(24) and Column (30)–(31) with capital and population controls, which shows whether GRP and industrial output were statistically comparable between the treatment group and control group. Further, Column (30)–(31) directly shows that the Solow residual held almost consistent between the two groups. It found that the introduction of control variables slightly reduces the difference at a level among the two groups’ common trends. Figure 1 presents more intuitively the results of output and output index at the constant price level (limited in the confidence interval at 95% significant level), which shows that there were not any expected treatment effects before 2003, and the treatment effect lasts for around three years from 2003 when the SARS epidemic broke out. 

As for consumption, we find two interesting and unexpected conclusions. On the one hand, during the whole year of the outbreak, there was neither a significant catastrophic decline nor over-expenditure. It means that although consumption was significantly hit during the tough period, it recovered soon and covered the loss during the epidemic. Consumers simply delayed their purchases but not cancelled. On the other hand, they did not buy more as “Retaliatory Consumption” after the epidemic. However, the retail sector of the economy was flourishing two years after the epidemic in the places where the epidemic hit severely, which promoted economic growth.

Lastly, we discuss the dynamics of government activities. Table 4 provides more clues about government activities. With respect to the current effect, it shows similar results to Table 3. Furthermore, Column (27) shows that the expansion of fiscal expenditure continued into the year after the epidemic but with a lower scale. Moreover, we find that fiscal income slightly decreased during the epidemic but significantly expanded two years after. It is reasonable to suspect that the upward movement was a symbol of the confirmation of economic recovery rather than the persistence of tax preference policies for the 2003 epidemic.

The mechanism analysis reveals that the economic impacts of the disease and relevant policies might be continuous and systemic. It cannot be ignored that there was indirect impact of the treatment effect on the economic system. There seems to be a strong link between investment and delayed changes in consumption. Therefore, to confirm the association and prove our guess, we construct a Panel-VAR system with three variables: consumption, investment and output with the data used above.

Table 5 presents the VAR model setting check. Table 5a reports the VAR lag order selection criteria using AIC, BIC, and HQIC, which recommend using a three-period lag VAR model. Next, Table 5b presents Block exogeneity Wald tests for each variable and Granger causality tests between each two. The results illustrate that no variables could be excluded from the VAR system; in other words, these three variables were systematically linked. Despite the result that investment is not Granger-caused by consumption or output, it is Granger-caused by an economic system that consists of the other two variables. Notice that consumption was Granger-caused by investment, output and the system of both during the sample period, which may support that the consumption expansion that appeared two years after the epidemic was due to investment and output growth during the infectious disease outbreak.

**Figure 2:**
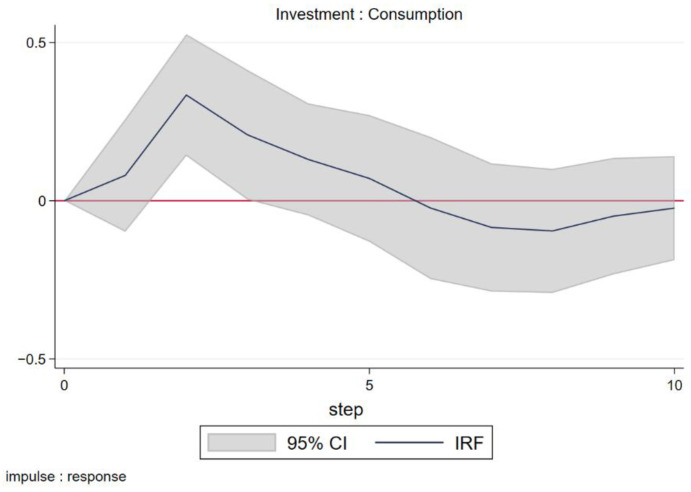
Impulse response of consumption to investment.

The impulse response in Figure 2 describes explicitly how consumption responds to the investment shock. As common sense would dictate, the response of consumption to the investment shock was positive and will last for several years. Not surprisingly, it took two years for the response to reach a peak, which coincides with the boom in consumption two years after the outbreak.

Using these clues, we may describe the whole story of the economic impact of the SARS epidemic in China in 2003. Like other natural disasters, the SARS epidemic struck several provinces in China without any economic signals and spread rapidly through labor flow. The government was forced to control the epidemic after thoroughly assessing the severity, danger and contagion of the infectious disease but still with limited information about how to cure the unknown disease. According to the different purposes, these policies could be roughly divided into two types: one type was to save lives mainly in the economic form of government purchase; the others were to save the economy in the form of taxes and fees discount. The policy focus gradually shifted from the former to the latter as the epidemic progresses. However, both the former and the latter were in the form of governmental capital transfer to the market. Similarly, some governments choose to issue new bonds or special bonds to control the epidemic, it is also another form of capital transfer, but the transfer is the government’s future capital, or in other words, the government’s credit. Like other expansionary fiscal policies, these policies increased aggregate demand in the short term, which led to the expansion of investment inducing economic growth. The excess investments promoted the retail market and then consumption through the production cycle. The stimulative effects of the policies on consumption reached their peak after two years, which led to a boost in output and fiscal revenue.

#### 3.3.3. Long-Term Impact of an Epidemic after the Reaction Mechanism

The mechanism of the epidemic’s impact on the economy has been clarified in the last subsection. An entire epidemic period starts from an unforeseen epidemic outbreak. It spreads undetected and is overlooked by the health department until its infections and risks have reached a level that cannot be ignored. Having collected enough information, the government would implement some interventions to control the epidemic spread and then some fiscal stimulus plan until the epidemic is under control. The second sub-period would begin at the end of the epidemic in a biological sense, but its economic impact would not disappear until the economic system has digested it.

Under this logic, the post-epidemic period would last for at least three years after the outbreak in our instance. To identify the long-term effect of the epidemic’s reaction mechanism, we hide the epidemic period from 2003, when the epidemic broke out, to 2005, when consumption recovered, to compare the difference between two steady states: pre-epidemic state and post-epidemic state, by using the following form with a standard DD model,

(9) $$y_{i,t}=\rho_{L}\cdot \;tre\;\cdot \;Post\;+X_{i,t}\theta +\eta_{i}+\eta_{t}+\varepsilon_{i,t}$$

where $\rho_{L}$ captures the long-term treatment effect of the mechanism, tre takes a similar meaning as above to distinguish the treatment and control group. Post is an indicator that takes the value of one during the post-epidemic period. Other variables have the same meaning as above, $X_{i,t}$ presents the logarithmic form of control variables and $\eta_{i}$,$\eta_{t}$ presents the provincial and time fixed effect. In order to enlarge our dataset as long as possible, we select the sample period from 1996 to 2016, but the samples during 2003–2005, when it was defined as the epidemic period, are hidden. The data source is the same as above, and the output and output index at a constant price are selected as dependent variables to eliminate the influence of price level.

Table 6, Panel A reports the long-term treatment effect of the epidemic reaction mechanism on output and the output index at a constant price. We also report it on output per capita in Panel B and the treatment effect on input factors, capital stock and population, with control variables in Columns (33) and (35) of Panel A while only capital stock is controlled in Panel B. All the dependent variables and control variables take logarithmic growth rate form in this analysis.

The results in Table 6 display that under almost the same factor input trend condition, all the output indicators presented significant negative results, showing a decline of around 2% on output and about 1% on per capita indicators. In this case, it is obvious that there was no significant permanent loss of capital or labor supply, suggesting the mechanism hit the economy in the affected area permanently. It is hard to dogmatically conclude that permanent damage was caused by crowding out effects, industrial transfer or other reasons between the two steady states before and after. However, it can be directly inferred that a decline occurred in resource allocation efficiency during the epidemic period.

## 4. Discussion

The SARS epidemic lasted for around 7 months in China from when the first case was found on Nov. 16th, 2002 to Jun. 24th, 2003 when the WHO removed mainland China from the epidemic area list. There is a large body of literature suggesting that the epidemic lead to economic loss in China while this study found strong and robust evidence suggesting that the SARS epidemic and its associated countermeasure policies boosted local output by around 4% and industrial production by around 5%. The evidence from city-level data provides a similar positive but smaller effect at about 3% but almost the same on industrial production at around 5%, suggesting that industry benefited from the epidemic and associated policies. The following three points that are different from previous research may lead to these counterintuitive results:

Initially, the more positive government activities related to the epidemic are considered in this study. Much attention was put on the epidemic itself, but limited attention was put on the economic impact of the health policies aimed at controlling the epidemic. Tanaka (2022) documented the policies designed to hedge the health or economic impact of epidemics [25]. There is some strong and robust evidence in this paper that proves that these policies, including public health policies and recovery plans, had expanded government consumption, which affected the production cycle similar to expansionary fiscal policies.

Secondly, the economic impact of the epidemic was dynamic and would last for at least 2 years. Rather than treating the epidemic only as a temporary shock on output, such as Beutels (2009) [4], the dynamic economic mechanism of the epidemic is concluded in this study. The expansion of government activities led to an increase in investment, which boosts consumption after the outbreak and hit the long-term economic efficiency after that. The mechanism may be hidden after the outbreak and go to the surface again while the investment caused by the policies transforms into consumption. The epidemic period in an economic sense may be longer than it in a biological sense depending on the length of the production cycle in the epidemic area.

Last but not least, the production process, especially the industries, respond to the epidemic shock and related policy shock most strongly. A direct idea could be to test that the worst losses had been in tourism or trade because the lockdown or travel restriction or even fear of infection may decrease the flow of passengers and cargo. However, the results of this study show a more general conclusion that the epidemic and policies stimulated economic growth in China in 2003, especially industrial growth. It is probably because tourism accounted for a little share of 2003 China’s GDP.

Limited to the research goal and constrained by length, this paper did not further explore the reasons for the long-term efficiency loss, which may be a part of clues in the economic mechanism of the epidemic. Microeconomic analysis and firm-level or household databases may reveal more details and clues about this problem in our future work.

## 5. Conclusions

This study assesses the short-term and long-term economic impact of the SARS epidemic and related interventions in China. The SARS epidemic in China is a typical example which is an epidemic that was controlled quickly by government interventions to test the economic impact of the epidemic and policy shocks, which may provide some practical implications in the post-pandemic period of COVID-19.

First of all, the policymakers should be concerned about not only the direct impacts of the epidemic but also the expansion of government activities. The recovery plan should be considered with a comprehensive assessment.

Secondly, the impacts of the epidemic and related policies will run under the water for 2 years. It is necessary to monitor various indicators of the economy and to make adjustments. Another suggestion from this finding is that 2 years, which may depend on the length of the production chain in different countries, is an appreciable duration to issue special bonds for raising money to hedge the impact of the epidemic while the fiscal income would grow.

## Figures and Tables

**Table 1:** Economic impact of the SARS epidemic in China.

	Provincial-Level			City-Level			
	Standard OLS		Bootstrap Error	Standard OLS			Common Support
	(1)	(2)	(3)	(4)	(5)	(6)	(7)
Panel A: Gross Regional Production (GRP)							
*did*	0.0435 ***	0.0374 ***	0.0374 ***	0.0192	0.0216 **	0.0275 **	0.0206 *
	(0.0141)	(0.0134)	(0.0133)	(0.0126)	(0.0107)	(0.0108)	(0.0106)
Observations	496	496	496	1657	1657	1655	1654
R-squared	0.7434	0.7707	0.7707	0.1607	0.3160	0.3348	0.4746
Panel B: Gross Regional Industrial Production (GRIP)							
*did*	0.0541 **	0.0479 *	0.0479 *	0.0431 *	0.0538 **	0.0594 **	0.0473 *
	(0.0263)	(0.0261)	(0.0245)	(0.0259)	(0.0240)	(0.0244)	(0.0254)
Observations	496	496	496	1,649	1,649	1,649	1,649
R-squared	0.7135	0.7221	0.7221	0.2372	0.3723	0.3766	0.4008
Time FE	YES	YES	YES	YES	YES	YES	YES
Prov. FE	YES	YES	YES	YES			
City FE					YES	YES	YES
Full Control		YES	YES			YES	YES

Notes: (1) Significant at *p* < 0.01 ***, *p* < 0.05 **, *p* < 0.1 * levels. (2) Standard errors are in parentheses, but it reports bootstrap standard error in Column (3) and robust standard error in Column (4)–(7). Matching samples are used in Column (7) with PSM by control variables. (3) Full Control indicates the use of control variables including capital input and labor input. Column (2)–(3) are controlled by the capital stock estimated by using the method of Shan (2008). Specifically, we use the control variables to estimate propensity score for matching and then test treatment effect without any control except fixed effects.

**Table 2:** Robustness Checks.

Panel A	Use 1996–2016			
	GRP	GRP Index	GRP Per Capita	GRP Index Per Capita
	(8)	(9)	(10)	(11)
*did*	0.0392 ***	0.0218 ***	0.0452 ***	0.0274 ***
	(0.0137)	(0.0067)	(0.0144)	(0.0074)
Observations	620	651	620	651
R-squared	0.7332	0.6653	0.7178	0.6278
**Panel B**	**Use 1999–2007**			
	**(12)**	**(13)**	**(14)**	**(15)**
*did*	0.0232 **	0.0111 **	0.0283 **	0.0156 ***
	(0.0108)	(0.0048)	(0.0114)	(0.0058)
Observations	279	279	279	279
R-squared	0.8053	0.8384	0.7846	0.7711

Notes: (1) Significant at *p* < 0.01 ***, *p* < 0.05 ** levels. (2) Standard errors are in parentheses. (3) All the dependent variables use their logarithmic difference form. (4) All the regressions in this table are controlled by year fixed effect and province fixed effect.

**Table 3:** Mechanism Identification.

	Consumption	Investment	Fiscal Expenditure	Fiscal Revenue	Fiscal Depict	Freight Traffic	Passenger Traffic
	(16)	(17)	(18)	(19)	(20)	(21)	(22)
*did*	−0.0135	0.0584 *	0.0614 **	−0.0397	0.2709 ***	−0.0183	−0.0235
	(0.0198)	(0.0322)	(0.0276)	(0.0310)	(0.0767)	(0.0719)	(0.1121)
Observations	372	372	372	372	371	372	372
R-squared	0.6298	0.5596	0.4673	0.4819	0.2313	0.1704	0.1961

Notes: (1) Significant at *p* < 0.01 ***, *p* < 0.05 **, *p* < 0.1 * levels. (2) Standard errors are in parentheses. (3) All the dependent variables use their logarithmic difference form. (4) All the regressions in this table are controlled by year fixed effect and province fixed effect. (5) The fiscal deficit is calculated by subtracting local fiscal revenue from local fiscal expenditures. (6) We lost 1 sample of financial deficit in 1998 of Fujian province because Fujian’s fiscal deficit was negative in 1997 while other provinces were positive during the sample period.

**Table 4:** Dynamic Mechanism Identification.

	GRP	GRIP	Consumption	Investment	Fiscal Expenditure	Fiscal Revenue	Fiscal Depict	GRP	GRIP
	(23)	(24)	(25)	(26)	(27)	(28)	(29)	(30)	(31)
…									
*prd_2001*	0.0126	0.0091	0.0002	0.0395	0.0218	0.0070	0.1040	0.0147	0.0149
	(0.0157)	(0.0343)	(0.0268)	(0.0440)	(0.0376)	(0.0421)	(0.1047)	(0.0153)	(0.0343)
*prd_2002*	0.0139	0.0203	0.0058	0.0589	0.0288	0.0332	0.0844	0.0136	0.0232
	(0.0157)	(0.0343)	(0.0268)	(0.0440)	(0.0376)	(0.0421)	(0.1047)	(0.0152)	(0.0342)
*did*	0.0363 **	0.0465	−0.0018	0.0979 **	0.1052 ***	−0.0124	0.3784 ***	0.0321 **	0.0456
	(0.0157)	(0.0343)	(0.0268)	(0.0440)	(0.0376)	(0.0421)	(0.1047)	(0.0153)	(0.0343)
*posd_2004*	0.0185	0.0398	0.0239	0.0681	0.0691 *	0.0577	0.1359	0.0143	0.0384
	(0.0157)	(0.0343)	(0.0268)	(0.0440)	(0.0376)	(0.0421)	(0.1047)	(0.0153)	(0.0342)
*posd_2005*	0.0286 *	0.0340	0.0611 **	0.0498	0.0480	0.0703 *	0.0726	0.0207	0.0270
	(0.0157)	(0.0343)	(0.0268)	(0.0440)	(0.0376)	(0.0421)	(0.1047)	(0.0153)	(0.0343)
…									
Observations	372	372	372	372	372	372	371	372	372
R-squared	0.7825	0.5994	0.6411	0.5660	0.4798	0.4942	0.2434	0.7978	0.6060
Control								Yes	Yes

Notes: (1) Significant at *p* < 0.01 ***, *p* < 0.05 **, *p* < 0.1 * levels. (2) Standard errors are in parentheses. (3) All the regressions in this table are controlled by year fixed effect and province fixed effect. (4) Due to limited space, only the counterfactual treatment effects in the two years before and after are reported in the table. In the unreported section, almost all the results are not significant, except that fiscal expenditures showed a significant treatment effect (at 95% significant level) in 1997 and 2000, fiscal deficits present positive significance in 1999 and 2000 (at 90% significant level).

**Table 5:** PVAR setting tests.

(a) PVAR Lag Order Selection Criteria				
Lag	AIC	BIC	HQIC	
1	−9.00	−7.77	−8.50	
2	−9.35	−7.90	−8.77	
3	−9.82 *	−8.12 *	−9.14 *	
4	−9.60	−7.59	−8.79	
5	−9.35	−6.95	−8.38	
**(b) PVAR Granger Causality/Block Exogeneity Wald Tests**				
Equation	Excluded	Chi-sq.	*p*-value	
Consumption	Investment	15.99	0.00	***
	Output	29.23	0.00	***
	ALL	83.33	0.00	***
Investment	Consumption	2.05	0.73	
	Output	7.19	0.13	
	ALL	21.34	0.01	***
Output	Consumption	10.64	0.03	**
	Investment	28.67	0.00	***
	ALL	62.39	0.00	***
H0: Excluded variable does not Granger-cause equation variable				

Notes: Significant at *p* < 0.01 ***, *p* < 0.05 **, *p* < 0.1 * levels.

**Table 6:** Long term economic impact of epidemic reaction mechanism.

	Output	Output	Output Index	Output Index
	(32)	(33)	(34)	(35)
Panel A: output				
treated post	−0.0280 ***	−0.0239 ***	−0.0120 ***	−0.0111 ***
	(0.0067)	(0.0056)	(0.0031)	(0.0027)
Obs	527	527	558	527
R-squared	0.7447	0.8203	0.6852	0.7768
Panel B: output per capita				
treated post	−0.0341 ***	−0.0311 ***	−0.0201 ***	−0.0184 ***
	(0.0070)	(0.0068)	(0.0034)	(0.0032)
Obs	527	527	558	527
R-squared	0.7312	0.7503	0.6513	0.7200
Control		YES		YES
Panel C: Factor Input				
	Capital Stock	Population		
	(36)	(37)		
treated post	−0.0133	0.0048		
	(0.0085)	(0.0037)		
Obs	527	527		
R-squared	0.3871	0.2733		

Notes: (1) Significant at *p* < 0.01 ***. (2) Standard errors are in parentheses. (3) Control indicates the use of control variables including capital stock and population in column (33) and only capital stock in column (35). (4) All the regressions in this table are controlled by year fixed effect and province fixed effect.

## Data Availability

Data is available from the authors upon reasonable request.
